# Functional Analysis of the Glucuronyltransferases GlcAT-P and GlcAT-S of *Drosophila melanogaster*: Distinct Activities towards the O-linked T-antigen

**DOI:** 10.3390/biom6010008

**Published:** 2016-01-06

**Authors:** Isabelle Breloy, Tilo Schwientek, Deborah Althoff, Marvin Holz, Tim Koppen, Angelika Krupa, Franz-Georg Hanisch

**Affiliations:** 1 Institute for Biochemistry II, Medical Faculty, University of Cologne, Joseph-Stelzmann Str. 52, Cologne 50931, Germany; althoffd@smail.uni-koeln.de (D.A.); marvin.holz@hhu.de (M.H.); TimKoppen@web.de (T.K.); akrupa@gmx.de (A.K.); franz.hanisch@uni-koeln.de (F.-G.H.); 2 Octapharma Biopharmaceuticals GmbH, Im Neuenheimer Feld 590, Heidelberg 69220, Germany; tilo.schwientek@octapharma.com

**Keywords:** glucuronyltransferases, *Drosophila melanogaster*, *N*-glycans, *O*-glycans, mass spectrometry, glycomics

## Abstract

The *Drosophila melanogaster* glucuronyltransferases dGlcAT-S and dGlcAT-P were reported to be expressed ubiquitously and results of *in vitro* activity assays indicate a functional redundancy. We analyzed both transferases *in vivo* and *in vitro* and could show significant differences in their activity towards *N*-and *O*-glycoproteins *in vivo*. While GlcAT-P is able to use N-linked *N*-acetyllactosamine chains and the O-linked T-antigen as a substrate to form non-sulfated HNK1- (GlcAβ1-3Galβ1-4GlcNAcβ1-) and glucuronyl-T-antigens *in vivo*, GlcAT-S adds glucuronic acid only to N-linked chains, thereby synthesizing only the non-sulfated HNK1-antigen.

## 1. Introduction

Despite the fact that *Drosophila melanogaster* has been used as a model organism for genetics since several decades, its application as a model organism for glycomic studies is still in its infancy. Yet, knowledge about glycan structures and glycosylation enzymes in *Drosophila* is constantly increasing. Previous studies on the glycome of the fruit fly *Drosophila melanogaster* have revealed restricted patterns of oligomannose N-linked sugar chains and simple mucin-type *O*-glycans dominated by the T-antigen (Galβ1-3GalNAcα-). More advanced studies applying novel sensitive MS-methods later revealed the existence of minor amounts of LacNAc chains on *N*-glycans, core 2-based *O*-glycans, short *O*-fucosyl- and *O*-mannosyl-glycan chains as well as an abundant modification of *O*- and *N*-glycans with terminal glucuronic or sialic acid [1,2,3].

While the terminal modification with sialic acid is abundant in the mammalian glycome, a modification with glucuronic acid seems to be largely restricted to *N*-glycans on cell adhesion molecules and on glycolipids. Mainly the HNK1-epitope (SO_4_-3GlcAβ1-3Galβ1-4GlcNAcβ-R) can be detected in neuronal tissue of mammals. It has been shown to be important for cell-cell and cell-substrate interactions especially during neuronal development [4,5].

A non-sulfated HNK1-epitope has first been detected immunologically on *N*-glycans of *Drosophila* [3]. Mass spectrometric evidence for terminal glucuronic acid on the T-antigen was first reported for *O*-glycans from Schneider S2 cells [2] and for *Drosophila* embryos on *O*-fucose glycans [1]. About 20%–30% of the *O*-glycans in *Drosophila* embryos are modified with glucuronic acid [1]. Despite this high percentage, a function of this modification has not been elucidated so far. Acidic sugars are known to play vital roles in a variety of glycan environments. Sialic acid e.g., fulfills important physiological functions in vertebrates (*i.e.*, in the formation of electrostatic cell shields, the exposure of blood-group-related antigens, *etc.*). In *Drosophila*, sialic acid occurs only in very small quantities synthesized by a small subpopulation of cells [6].

The modification of most of *Drosophila’s* glycan types with glucuronic acid leads to the assumption that glucuronic acid plays an important role in the fruit fly, which may be comparable to the role of sialic acid in mammals. To unravel the functional context of glucuronic acid in *Drosophila*, the elucidation of HNK-1 (GlcAβ1-3Galβ1-4GlcNAcβ-R) and glucuronyl-T (GlcAβ1-3Galβ1-3GalNAcα-R) biosynthesis are of utmost importance. Three genes homologous to the mammalian β1-3glucuronyltransferases have been found in *Drosophila*. *In vitro* studies of the recombinantly expressed enzymes have shown that each isoform exhibits discrete functions in glycosaminoglycan and glycolipid synthesis [7].

The *Drosophila* transferase GlcAT-I is highly homologous to the human transferase GlcAT-I. Both were shown to be specific for the addition of GlcA to the core region of glycosaminoglycan (GAG) chains. They exhibit no activity towards the glycan chains on glycoproteins or glycolipids. Mammalian GlcAT-P and GlcAT-S, on the other hand, have rigid substrate specificities towards the Galβ1-4GlcNAc epitope, synthesizing the non-sulfated HNK1-epitope (GlcAβ1-3Galβ1-4GlcNAcβ1-) on glycoproteins and glycolipids. GlcAT-P and GlcAT-S are distributed widely in different regions of adult mouse brain. GlcAT-P expression correlates with non-sulfated HNK1-epitope staining, but GlcAT-S may also act on other targets [8]. While the expression of these enzymes in mammals is limited to neuronal tissue [9] and kidney [10], the *Drosophila* orthologues are expressed ubiquitously. dGlcAT-S and dGlcAT-P are ubiquitously expressed during all developmental stages of the fly. *In vitro*, the enzymes exhibit broad substrate specificity with formation of N-linked HNK-1 and O-linked glucuronyl-T antigens [7].

In this study, we confirm earlier results [7] showing that dGlcAT-S and dGlcAT-P can transfer GlcA to the O-linked T-antigen as well as to N-linked LacNAc-chains *in vitro*, however, with slightly different activity profiles. Recombinant overexpression in S2-cells reveals an increase in glucuronyl-T-antigen expression induced by dGlcAT-P. *In vivo*, only dGlcAT-P accepts the O-linked T-antigen as substrate and forms the glucuronyl-T antigen, while both transferases are able to synthesize the non-sulfated HNK1-epitope on *N*-glycans.

## 2. Results and Discussion

### 2.1. Establishing Epitope Specificities of Monoclonal Anti-GlcA Antibodies

MAb M6749 has previously been described [9,10,11] as detecting the non-sulfated as well as the sulfated HNK1-epitopes ((SO4)-3GlcAβ1-3Galβ1-4GlcNAcβ1-) [9,10,11]. In search for antibodies defining the glucuronyl T-antigen, we characterized MAb 114-2G11-A (kindly provided by Ron Hokke, LUMC Leiden, The Netherlands) as binding to terminal β1-3-linked glucuronic acid independently of the glycan type (N- or O-linked chains). We tested the specificities of mAbs M6749 and 114-2G11-A by immunoblot using *in vitro* glucuronylated asialofetuin, α1-acid glycoprotein (A1AGP) and recombinant MUC1-VH as antigens (Figure S1). While the *N*- and *O*-glycosylated protein asialofetuin and the *N*-glycoprotein A1AGP were detected by both antibodies, *O*-glycoprotein MUC1-VH was only recognised by mAb 114-2G11-A, confirming that mAb M6749 is specific for the non-sulfated HNK1-epitope while mAb 114-2G11-A generally detects terminal β1-3-linked glucuronic acid modifications. The different specificities were additionally verified [1] by β-glucuronidase digestion of the antigen which leads to a loss of antigen binding by both antibodies, and [2] by PNGaseF digestion which affected only the binding of mAb M6749 (Figure S1).

### 2.2. Recombinant Expression of dGlcAT-P and dGlcAT-S in Drosophila S2-Cells

To investigate the β1-3-glucuronyltransferase isoforms *in vivo* und *in vitro*, we generated vectors for recombinant expression of dGlcAT-P and dGlcAT-S in cultivated *Drosophila* Schneider 2 (S2) cells. These include constructs that (1) intracellularly express the full-length glycosyltransferases (containing the cytoplasmic domain, transmembrane domain, stem region, and the catalytic domain) and (2) vectors that, *via* deletion of the cytoplasmic tail and the transmembrane domain, encode for secretory fusion proteins comprising a shortened stem region and the catalytic domain of the enzymes.

**Figure 1:**
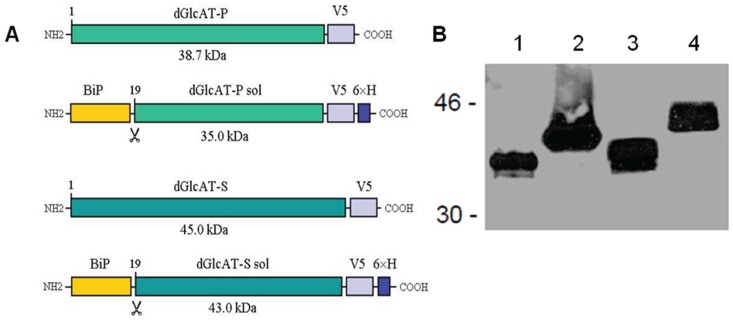
(**A**) Recombinant glucuronyltransferase fusion proteins expressed in *Drosophila* cells. Plasmids encoding intracellular (dGlcAT-P, dGlcAT-S) and secreted (dGlcAT-P sol, dGlcAT-S sol) fusion proteins with C-terminal *V5* and *6 × His*-*Tags* were generated. *BiP*, Hsc70-3 signal sequence; *dGlcAT*, *Drosophila* β1-3-glucuronyltransferase; *V5*, Paramyxovirus SV5-antigen. (**B**) Western blot analysis of cell supernatants and cell lysates of transfected Drosophila S2 cells with mAb anti-V5. **1**, cell culture supernatant of S2-cells expressing dGlcAT-P sol; **2**, culture supernatant of S2-dGlcAT-S sol; **3**, cell lysate of S2-dGlcAT-P; **4**, cell lysate S2-dGlcAT-S.

The complete cDNA of the transferases was amplified by PCR, fused with a V5-tag for immunodetection and cloned into a constitutive *Drosophila* expression vector. The expressed full-length enzymes were analysed in S2-cell lysates. For generation and expression of secreted glycosyltransferases, a truncated version of the enzymes was cloned into an inducible *Drosophila* expression vector and fused with the V5- and a 6× His-tag for purification of the enzymes by Ni-NTA affinity chromatography. Secretion into the culture medium was facilitated by an *N*-terminal fusion of the *Drosophila* BiP/Hsc70-secretion signal. The secreted fusion proteins dGlcAT-Psol and dGlcAT-Ssol were purified from the S2-cell-culture supernatant (Figure 1).

### 2.3. In Vivo Glucuronylation Studies

#### 2.3.1. Glucuronylation of *O*-linked Chains

**Figure 2:**
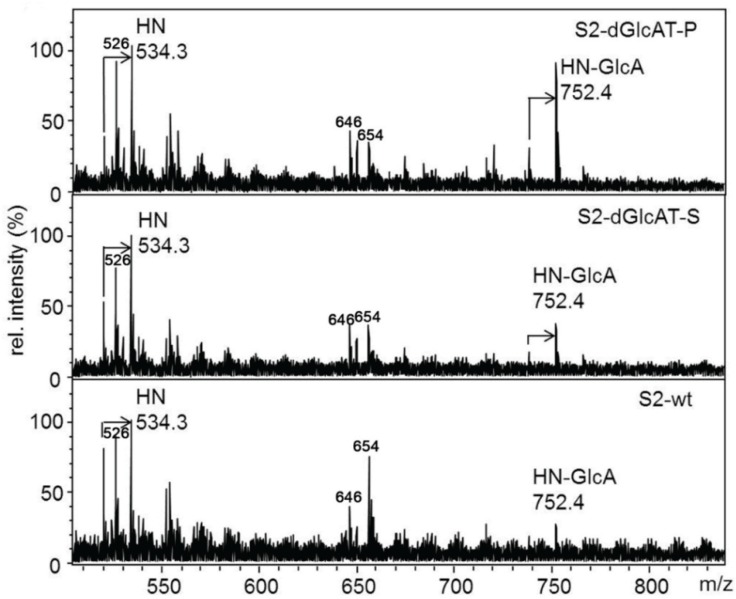
MALDI-MS of permethylated *O*-glycans from S2-cell lysates. In comparison to wt cells (lower panel), S2 cells overexpressing dGlcAT-P (upper panel) show an increase in non-sulfated glucuronyl-T-antigen expression (HN-GlcA + Na, *m*/*z* 752) relative to the precursor T-antigen (HN+Na, *m*/*z* 534), while no difference is observed in dGlcAT-S overexpressing cells (middle panel). The arrows indicate signals corresponding to mono-undermethylated proton adducts (MNa-36). Other mass signals in the range up to 1000 Da refer to matrix-derived ions. Short notation of glycan structures: H, hexose, N, *N*-acetylhexosamine, GlcA, glucuronic acid.

The *O*-glycome of S2-cells transfected with vectors encoding full-length dGlcAT-S or dGlcAT-P were analyzed and compared to the *O*-glycome of S2 wt cells. Due to the high activity of endogenous glucuronyltransferases, only minor quantitative differences in glycan glucuronylation were observed by MALDI-MS of the permethylated *O*-glycan chains. In comparison to S2 wt-cells, overexpression of dGlcAT-P led to a higher expression of the glucuronyl-T antigen as observed by an increase in the ratio of the glucuronyl-T (*m*/*z* 752) relative to the T antigen (*m*/*z* 534). Overexpression of dGlcAT-S did not induce significant changes in the fraction of mucin-type *O*-glycans of S2 cell proteins (Figure 2).

Immunostaining of Western blots with mAb114-2G11-A revealed a strong increase of GlcA expression on proteins in the mass range around 70 kDa. However, this strong increase was confined to dGlcAT-P overexpressing cells whereas S2-cells overexpressing dGlcAT-S did show only slightly increased protein glucuronylation in this mass range. PNGaseF digestion resulted in a shift to lower masses, but not to changes of the staining pattern and signal intensities, which might be explained by the assumption that most of the GlcA-epitopes in S2-cells are bound to *O*-glycans (Figure S2).

#### 2.3.2. Glucuronylation of N-Linked Chains

**Figure 3:**
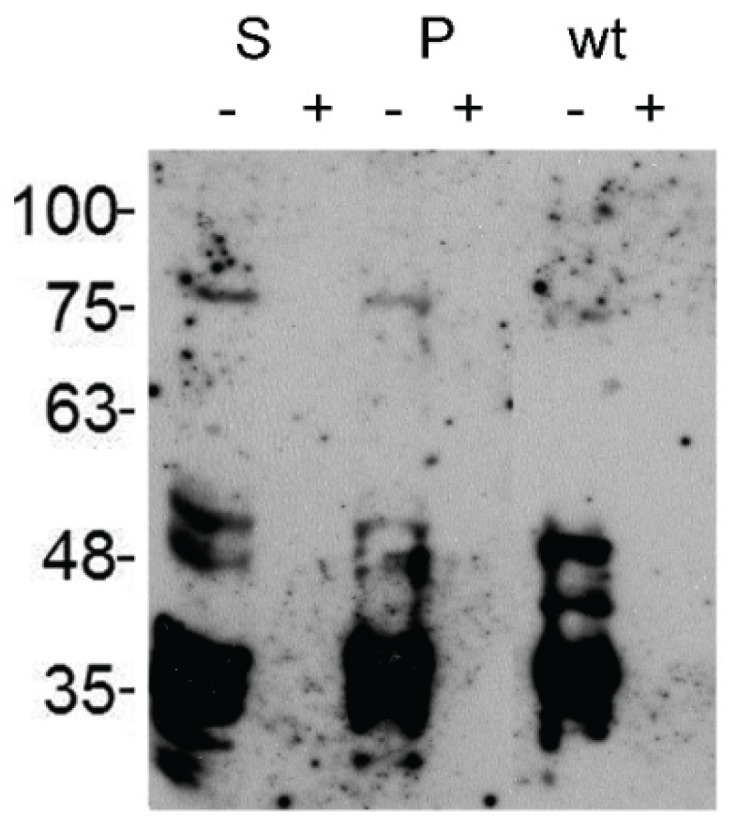
Western blot of S2 cell lysates, immunostained with non-sulfated HNK1-epitope specific mAb M6749. The signals appear exclusively after prolonged exposure times and only minor differences can be observed between dGlcAT-S (S), dGlcAT-P (P) transfected and wildtype (wt) cells. All signals disappear completely after PNGaseF digestion (+) confirming the localisation of the non-sulfated HNK1-epitope on *N*-glycan chains.

The attempt to detect glucuronylated *N*-glycans by MALDI mass spectrometry revealed only signals with trace intensities rarely exceeding signal-to-noise ratios of >2. We could detect non-sulfated HNK1-modified proteins from S2 cell lysates on immunoblots with the non-sulfated HNK1-specific mAb M6749 combined with chemiluminescence detection, however, only after prolonged exposure times (Figure 3). The signals disappeared completely after PNGaseF-digestion, confirming the localization of the non-sulfated HNK1-epitope on *N*-glycans. There was almost no quantitative nor qualitative difference in the staining pattern of wildtype and dGlcAT-S or dGlcAT-P overexpressing cells.

Taken together, the above results revealed that, in the transfected *Drosophila* S2-cells, most of the glycoprotein-modifying glucuronic acid is bound to *O*-glycan chains, while only minor amounts are bound to *N*-glycans. This agrees with the low expression of complex *N*-glycan chains in *Drosophila melanogaster* which are a prerequisite for the formation of the non-sulfated HNK1-epitope. While overexpression of dGlcAT-P in S2-cells led to a significant increase of the glucuronyl-T antigen, overexpression of dGlcAT-S-cells did not cause any detectable differences in the cellular glycome. As previously speculated for the role of GlcAT-S in mouse [8], *Drosophila* GlcAT-S may be active in *Drosophila* also on targets other than glycoproteins.

#### 2.3.3. Proteomic Identification of Glucuronic Acid-Modified Proteins

**Figure 4:**
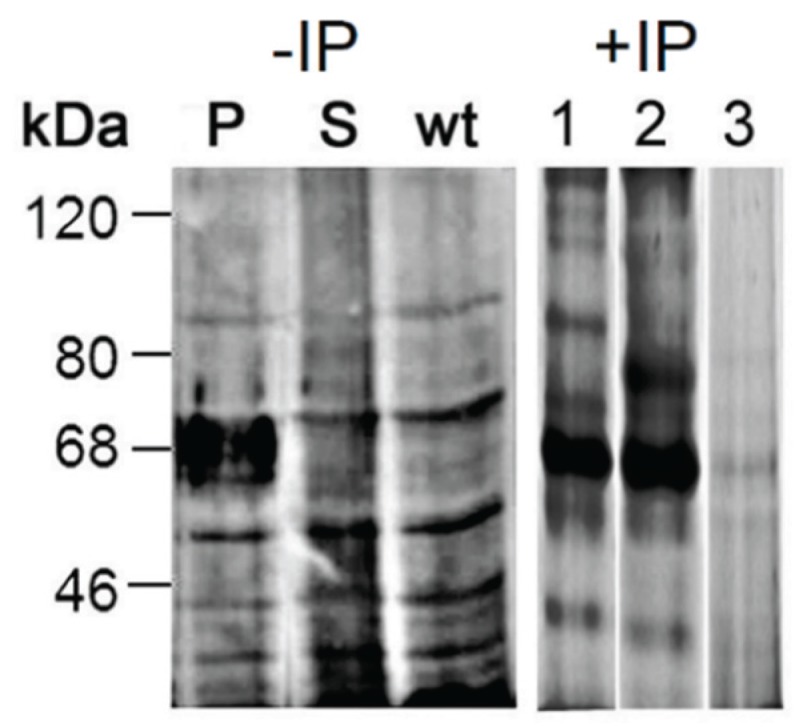
Immunostaining of Western blots with mAb 114-2G11-A (anti-GlcA) of cell lysates from S2-wt and dGlcAT-P (P) or dGlcAT-S (S) overexpressing cells reveals a strong increase of GlcA-epitopes in cells overexpressing GlcAT-P in the mass range around 60–70 kDa. A silver-stained SDS-PAGE of proteins immunoprecipitated with anti-GlcA specific mAb 144-2G11-A (lane 1) from S2-dGlcAT-P cell lysates (IP-P) shows a comparable pattern with most intense protein staining in the mass range of 60–70 kDa (arrow). Immunoprecipitation with the anti-T antibody HH8 (lane 2) leads to a partial overlap of precipitated proteins, whereas a nonrelevant IgM antibody (lane 3) did not reveal comparable protein enrichment in the respective mass range, indicating that most of the immunoprecipitated protein in lanes 1 and 2 had specifically bound to the carbohydrate-specific antibodies.

As immunostaining of Western blots with mAb114-2G11-A revealed a strong increase of GlcA expression on proteins in the mass range around 70 kDa in dGlcAT-P overexpressing cells (see 2.3.1) we excised a gel slice in the respective mass range from SDS-gel lane containing proteins of an S2-dGlcAT-P cell lysate and identified these by mass spectrometric proteomics. Identification was based on LC-ESI-MS/MS data of the tryptic peptides and Mascot searches in *Drosophila melanogaster* entries within the NCBI database. In the mass range of around 60–70 kDa, we detected a series of molecular chaperones (Table 1 and Table S1). To verify that these proteins are indeed glycosylated, we immunoprecipitated the same lysate with mAb 114-2G11-A. The precipitated proteins were visualized by silver staining of an SDS-PAGE and the staining pattern was highly comparable to the immunoblot of an S2-dGlcAT-P cell lysate with mAb 114-2G11-A (Figure 4, lane 1, Table 1, and Table S1). When using an anti-T antibody (HH8) protein enrichment was visible in the same mass range, but also distinct protein staining was observed (Figure 4, lane 2). The negative control generated by using a nonrelevant IgM revealed weak protein staining in the mass range of 60–70 kDa indicating that most of the immunoprecipitated protein in lanes 1 and 2 was specifically bound by the carbohydrate-specific antibodies (Figure 4, lane 3).

Applying mass spectrometric proteomics on the immunoprecipitated proteins, we identified the same molecular chaperones as the major components as in the gel slices of the mass range 60–70 kDa (Table S1). Other proteins with high Mascot scores, like bacterial effector Vopl or actin, correspond to co-precipitated species that potentially are bound by the chaperones.

**Table 1:** Potentially *O*-glycosylated proteins which were highly modified with GlcA after overexpression of dGlcAT-P in S2-cells. Proteins within a mass range of 60–70 kDa were intensively stained by mAb 114-2G11-A and therefore excised from an SDS-gel and identified by LC-ESI-MS/MS (experiment “gel”) The same lysates was immunoprecipitated with mAb 114-2G11-A and identified likewise (experiment “IP”). (ER: endoplasmatic reticulum, EC: extracellular, N: nucleus, CP: cytoplasm, MI: mitochondrium, MT: microtubule, M: membrane).

Protein	Identified in Experiment IP (+/−)	Reported Localization	Accession No. (NCBI)
calnexin	+	M, ER	gi|2213427
CD98 heavy chain	+	M	gi|17945866
CG2918, HSP70 family	+/−	EC, M	gi|20128923
ERp60	+/−	ER	gi|45551086
glycoprotein 93	+/−	EC	gi|21357739
heat shock protein 60	+/−	MI	gi|33636453
heat shock protein 83	+/−	CP	gi|17647529
heat shock protein cognate 1	−	MT	gi|17647515
heat shock protein cognate 4	+/−	N, CP, EC	gi|17737967
heat shock protein cognate 71	+/−	ER	gi|157667
heat shock protein cognate 72	+/−	ER, EC	gi|157658
Hexosaminidase 2	+	M, EC	gi|17933586
Inos	+/−	CP	gi|17137626
oligosaccharide transferase	+	M	gi|19922486
peptidase S28	+	EC, M	gi|20129649
protein disulfide isomerase	+/−	ER, EC	gi|17647799
Scavenger receptor class C	+	M	gi|984515
Ugt58Fa glycosyltransferase	+	M	gi|22024248
Ugt86Da glycosyltransferase	+	M	gi|21357701
vacuolar H[+]-ATPase	+	M, CP	gi|17136796

Proteins were identified prior to IP (−), after IP (+) or in both experimental settings (+/−).

**Figure 5:**
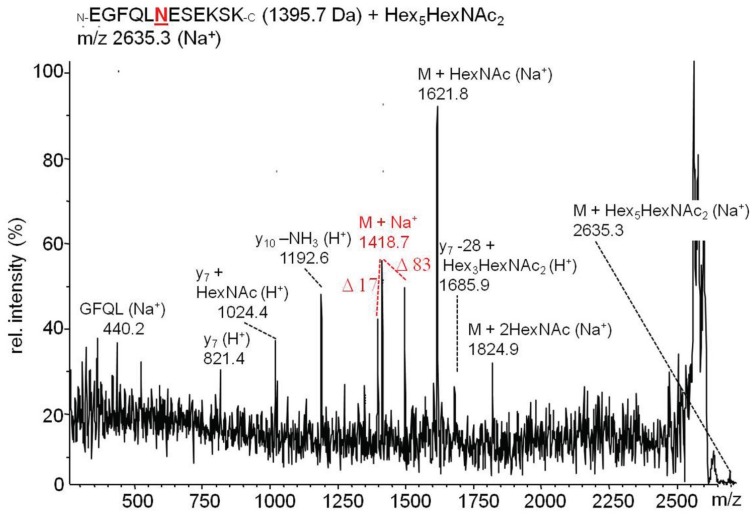
Post-Source-Decay MALDI-MS/MS spectrum of the glycopeptide EGFQLNESEKSK (1395.7 Da) from the molecular chaperone glycoprotein 93 modified with an *N*-glycan chain (Hex5HexNAc2, H5N2). We identified a series of y-ions, partially modified with *N*-glycans linked to asparagine within the consensus site NES. The unglycosylated peptide was identified by a triplet of signals representing the peptide mass as sodium adduct accompanied by signals at −17 Da and + 83 Da.

Analysis of the tryptic peptides after glycopeptide enrichment by MALDI-MS/MS in the Post-Source Decay mode revealed several glycopeptides which could be assigned to tryptic cleavage products from the identified chaperones (example shown in Figure 5).

It has already been reported that molecular chaperones in *Drosophila* can be secreted into the extracellular space [12]. ER-localized heat-shock proteins, disulfide isomerases and inositol phosphatases have previously been reported as *O*-glycosylated in “simple cells” (S2-KO cell models without the ability of core-GalNAc elongation) and proteins from the heat-shock family 70 have already been identified as *O*-glycoproteins in *Drosophila* [13,14,15].

### 2.4. In Vitro-Analysis of the Recombinantly Expressed Glucuronyltransferases

The efficient secretion of the soluble fusion proteins with a C-terminal 6× His-Tag facilitated the purification of enzymatically active β3-glucuronyltransferases *via* Ni-chelate affinity chromatography.

The activity of the purified soluble enzymes dGlcAT-P sol and dGlcAT-S sol as beta1-3 glucuronyltransferases was tested by *in vitro*-assays using low molecular weight acceptors. Purified dGlcAT-P sol and dGlcAT-S sol displayed high activity with LacNAc (Galβ1-4GlcNAc-benzyl) as well as with the T-antigen conjugate Galβ1-3GalNAc-benzyl as acceptor for glucuronic acid transfer. In an earlier study by Kim *et al.*, 2003 [7], several synthetic glycan targets as well as asialoorosomucoid were tested as acceptor substrates. To provide evidence for the activity on glycoproteins, we tested our recombinant transferases on the asialo-form of bovine 2-HS-glycoprotein (asialo-fetuin). This model protein carries terminal LacNAc (Galβ1-4GlcNAc-R) on *N*-glycan chains as well as the O-linked T-antigen (Galβ1-1GalNAc-R) that both are potential acceptor structures. The formation of glucuronylated *N*- and *O*-glycans on asialo-fetuin was verified immunologically and by mass spectrometry (MALDI-MS/MS).

#### 2.4.1. *In Vitro*-Synthesis of the Non-Sulfated HNK1-Epitope

**Figure 6:**
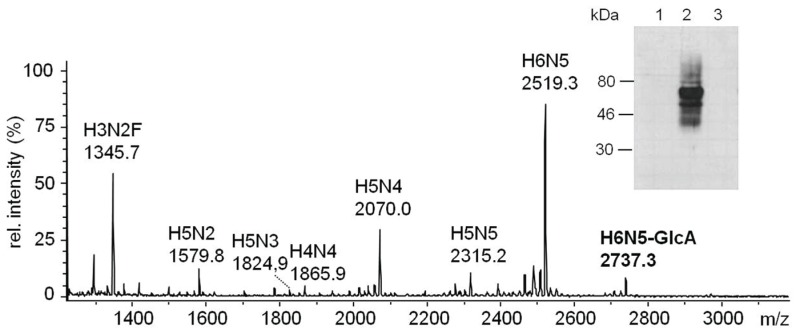
Analysis of the non-sulfated HNK1-antigen produced by *in vitro* glucuronylation of asialofetuin with dGlcAT-P sol. The MALDI-MS spectrum of the permethylated *N*-glycans shows a mono-glucuronylated sugar at *m*/*z* 2737. This was immunochemically verified by applying GlcA-fetuin (3 µg/lane) on a Western blot using the anti-HNK1 antibody M6749 (insert). The signal disappears after PNGaseF and β-glucuronidase digestion, verifying β1-3 linked GlcA on *N*-glycans. 1: GlcA-modified fetuin + PNGaseF; 2: GlcA-modified fetuin; 3: GlcA-modified fetuin + β-glucuronidase. Short notation of glycan structures: H, hexose; N, *N*-acetylhexosamine; F, fucose; GlcA, glucuronic acid.

Western blots of glucuronylated fetuin with mAb M6749 (anti-HNK-1, see 2.1) revealed evidence for the *in vitro* synthesis of non-sulfated HNK1-antigen by soluble dGlcAT-P and dGlcAT-S. The sensitivity of the anti-HNK1 signal to β-glucuronidase digestion demonstrated that the added glucuronic acid was β-linked. Digestion with PNGase F revealed the localization of the epitope on N-linked glycans of the *in vitro* glucuronylated protein (Figure 6). Next, the *N*-glycans of GlcA-fetuin were structurally analysed by MALDI-MS. The signal of a complex-type *N*-glycan chain at *m*/*z* 2738 (H6N5-GlcA, (M + Na^+^)), derived by glucuronylation of the dominant tri-antennary *N*-glycan with M + Na^+^ at *m*/*z* 2519 (H6N5), was detectable in assays with both transferases (Figure 6). The MALDI-MS spectrum of asialo-fetuin, glucuronylated with dGlcAT-S sol contained an additional signal at *m*/*z* 2955 which corresponds to the addition of two GlcA-residues to the complex *N*-glycan chain (Figure S3). The fine structure of the *N*-glycans of *in vitro* glucuronylated fetuin was verified by MS/MS (Figure S4).

#### 2.4.2. *In Vitro*-Synthesis of the Glucuronyl-T Antigen

**Figure 7:**
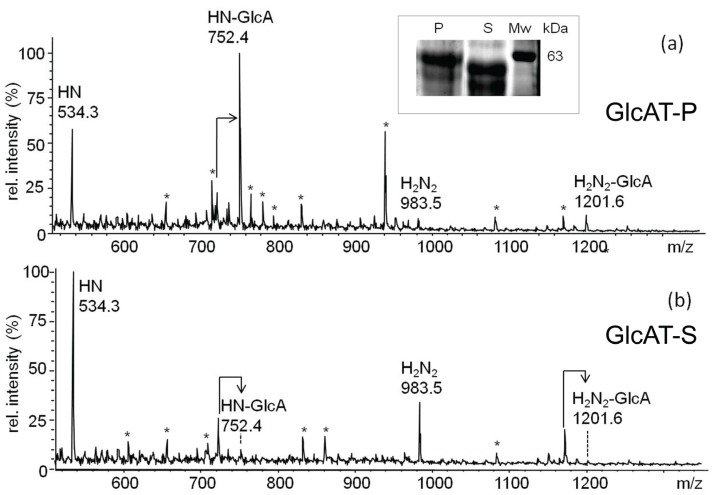
(**a**) MALDI mass spectrometric analysis of methylated *O*-glycan alditols derived from *in vitro* glucuronylated asialo-fetuin (dGlcAT-P sol). The mass spectrum reveals glucuronylated mucin-type glycan chains at *m*/*z* 752 (HN-GlcA, core 1) and 1201 (H2N2-GlcA, core 2). (**b**) MALDI mass spectrometric analysis of methylated *O*-glycan alditols derived from *in vitro* glucuronylated asialo-fetuin (dGlcAT-S sol). The mass spectrum shows the same pattern of glycan alditols as in (**a**), but the ratio of glucuronylated *O*-glycan chains *vs.* non-glucuronylated chains is significantly lower. (*: matrix signals and non-identified contaminants) The insert shows a coomassie-stained SDS-Gel of asialofetuin glucuronylated *in vitro* by dGlcAT-P (lane P) or dGlcAT-S (lane S). The difference in the apparent molecular masses corresponds to the higher activity of dGlcAT-P towards *O*-glycans as shown in (**a**,**b**). Short notation of glycan structures: H, hexose; N, *N*-acetylhexosamine; GlcA, glucuronic acid.

The formation of the glucuronyl-T antigen by dGlcAT-S and dGlcAT-P on asialo-fetuin *in vitro* was confirmed by Western blot using the terminal GlcA-specific mAb 114-2G11-A. The localisation of glucuronic acid on *O*-glycan chains was proven by PNGase F digestion that resulted in a mass shift of the protein, but not in a loss or decrease of staining intensity (Figure S1). The *O*-glycans released from *in vitro* glucuronylated fetuin were structurally analysed by MALDI-MS/MS. The glucuronylated *O*-glycans correspond to the mass signals at *m*/*z* 752 (HN-GlcA) and *m*/*z* 1201 (H2N2-GlcA) (Figure 7). The structure of HN-GlcA was verified by MS/MS (Figure S5).

The results obtained in the *in vivo* studies and the *in vitro* analysis indicated a higher activity of dGlcAT-P sol towards *O*-glycans, which can be confirmed by comparison of the molecular mass shifts of asialo-fetuin, glucuronylated *in vitro* by dGlcAT-P sol or dGlcAT-S sol (Figure 7). Analysis of the protein by SDS-PAGE revealed a higher molecular mass of fetuin when glucuronylated with dGlcAT-P, which is in accordance with mass spectrometric data of the mucin type *O*-glycans after *in vitro*-glucuronylation, revealing higher relative amounts of glucuronylated glycans after glucuronylation with dGlcAT-P compared to dGlcAT-S (Figure 7a,b).

### 2.5. Recombinant Expression of dGlcAT-P and dGlcAT-S in CHO-Lec2-Cells

The complete cDNA of dGlcAT-P and dGlcAT-S was cloned into the mammalian expression vector pCDNA3.1 for recombinant expression of the full-length transferases in CHO-Lec2 cells. These mutant CHO cells are deficient in the CMP-NeuAc transport into the Golgi lumen; therefore their N- and O-linked glycan chains cannot be modified with sialic acid *in vivo*. As a consequence, the cells expose terminal *N*-acetyllactosamine and other non-sialylated epitopes as potential GlcA-acceptors on glycoproteins and glycolipids. This makes the Lec2 mutant a suitable cell model for testing the efficiency and specificity of exogenous elongating glycosyltransferases *in vivo* avoiding the influence of endogenous background activities [16]. Expression of the *Drosophila* glucuronyltransferases with a C-terminal V5-tag allowed their immunochemical localization to the Golgi of Lec2 cells (Figure S6). All other experiments were performed with the non-tagged enzymes to rule out a possible influence of the tag on their enzymatic activity. *De novo* formation of the glucuronyl-T antigen *in vivo* was detected by mAb 114-2G11-A and of the non-sulfated HNK1-antigen by mAb M6749, as before.

#### 2.5.1. Coexpression of Glycosylation Probes in CHO-Lec2-Cells

To analyse changes in the glycome induced by recombinant expression of dGlcAT-P or dGlcAT-S in CHO-Lec2 cells, an *O*- and *N*-glycosylated fusion protein based on human nidogen-1 G1-G2 domains (Nid1, described in [17]) or on human α-dystroglycan (hDG5, described in [18]) was cotransfected with the heterologous transferases and purified from the cell culture supernatant by Ni-chelate affinity chromatography. Subsequently, the *O*- and *N*-glycan profiles were analysed immunologically and by MALDI-MS/MS with emphasis on a modification with glucuronic acid. In the context of *O*-glycosylation studies, the hDG5 probe was chosen as this glycoprotein model carries considerably more O-linked chains.

#### 2.5.2. *In Vivo* Analysis of dGlcAT-P

**Figure 8:**
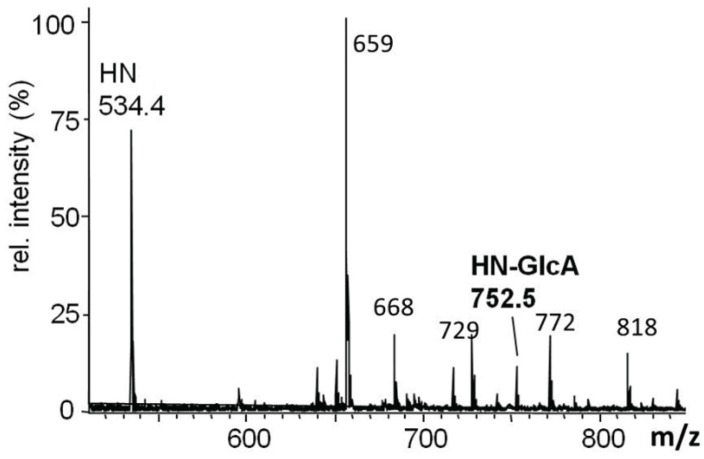
MALDI-MS spectrum of the permethylated *O*-glycan alditols derived by reductive β-elimination from hDG5 coexpressed with dGlcAT-P in CHO-Lec2 cells. An intense signal at *m*/*z* 752 (HN-GlcA + Na^+^) revealed a glucuronylation of the T-antigen (HN + Na^+^). Non-labeled signals in the mass range up to *m*/*z* 1000 are matrix-derived signals. Short notation of glycan structures: H, hexose; N, *N*-acetylhexosamine.

The glycans from *O*-glycoprotein hDG5 coexpressed with dGlcAT-P in CHO-Lec2-cells were analysed by MALDI-MS of the permethylated *O*-glycans released from the protein by beta-elimination. An intense signal at *m*/*z* 752 (HN-GlcA + Na+) revealed a glucuronylation of the T-antigen (HN + Na+) which demonstrated the *in vivo* function of the fly enzyme in the heterologous Lec2 hamster cell system (Figure 8).

**Figure 9:**
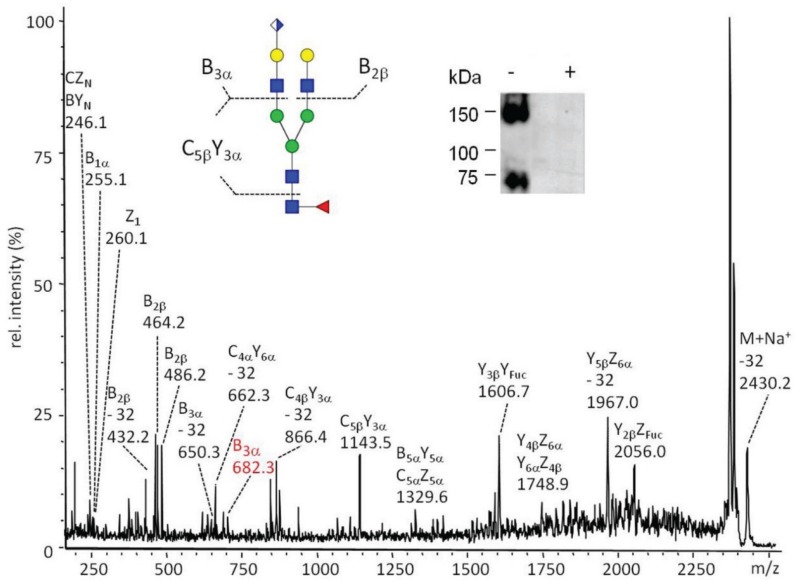
MALDI-MS/MS spectrum of a permethylated *N*-glycan chain bearing the non-sulfated HNK1-epitope at *m*/*z* 2430 (M + Na^+^), derived by PNGAseF-digestion of Nid1 coexpressed with dGlcAT-P in CHO-Lec2 cells. The glucuronylated glycan as well as part of the fragments were detected with a mass incremental loss of 32 Da, corresponding to the elimination of methanol. The presence of terminal GlcA is indicated by B series ions (B3α and B3α-32) and by double fragmentation ions (B3αY5α).

The *N*-glycans of nidogen-1 G1-G2 coexpressed with dGlcAT-P were analysed by Western blot using mAb M6749. In this case, the signals were sensitive to PNGaseF revealing a modification of the Nid1 *N*-glycan chains with the HNK1-epitope. The corresponding glycan structure could be detected by MALDI-MS of the permethylated *N*-glycan chains removed from the protein by PNGaseF-digestion (Figure S7). The fine structure of the non-sulfated HNK1-epitope at *m*/*z* 2430 (M + Na^+^) was verified by MALDI-MS/MS (Figure 9).

The insert shows a Western blot with *N*-glycan-specific mAb M6749 before (−) and after (+) PNGase F digestion. The PNGase F-sensitivity of the mAb M6749 signal confirmed the modification of *N*-glycan chains with glucuronic acid leading to the *in vivo* synthesis of the non-sulfated HNK1-epitope. Nid1 was detected as a monomer and dimer as concluded from the apparent molecular masses of 75 and 150 kDa and gel-based mass spectrometric proteomics.

#### 2.5.3. *In vivo* Analysis of dGlcAT-S

*In vivo* glucuronylation of hDG5 and nidogen-1 G1-G2 in CHO-Lec2 was also observed upon coexpression with full-length dGlcAT-S.

**Figure 10:**
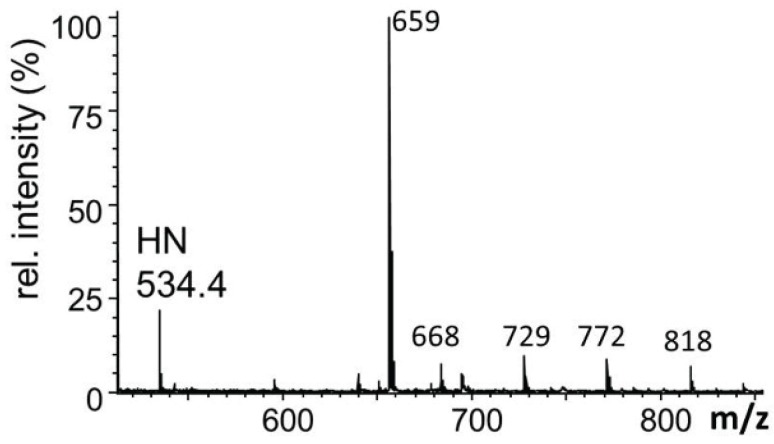
MALDI-MS spectrum of the permethylated *O*-glycan alditols derived by reductive β-elimination from hDG5 coexpressed with dGlcAT-S in CHO-Lec2 cells. No glucuronylation of *O*-glycans was observed. Non-labeled signals in the mass range up to *m*/*z* 1000 are matrix-derived signals or derived from a polyhexose impurity. Short notation of glycan structures: H, hexose; N, *N*-acetylhexosamine.

In contrast to hDG5 coexpressed with dGlcAT-P (see above), the glucuronyl T-antigen could not be detected in dGlcAT-S cotransfected cells by MALDI-MS/MS analysis of the permethylated *O*-glycan chains (Figure 10). This led to the conclusion that only *N*-glycans of the protein were glucuronylated by dGlcAT-S.

**Figure 11:**
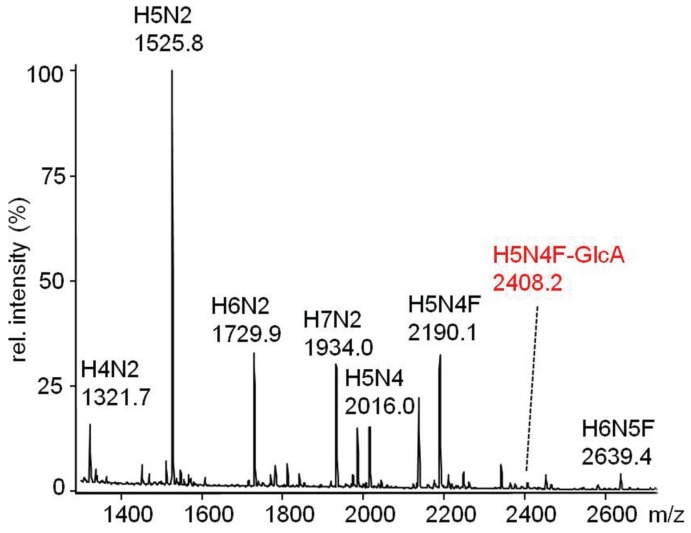
MALDI-MS spectrum of the permethylated *N*-glycans of Nid1, coexpressed with dGlcAT-S in CHO-Lec2 cells. The signals were detected as molecular ions M+Na^+^ -54, corresponding to a loss of sodium-methylate. The non-sulfated HNK1-epitope carrying glycan (H5N4F-GlcA) was detected at *m*/*z* 2408. The structure was verified by MS/MS. Short notation of glycan structures: H, hexose; N, *N*-acetylhexosamine; F, fucose; GlcA, glucuronic acid.

The *N*-glycans of nidogen-1 G1-G2 coexpressed with dGlcAT-S were analysed by Western blot using the mAb M6749 as described above (see 2.5.2). As already observed for Nid1/dGlcAT-P, the signal was sensitive to PNGase F, revealing a modification of the *N*-glycans with the non-sulfated HNK1-epitope. The corresponding glycan structure could be detected by MALDI-MS of the permethylated *N*-glycan chains released from the protein by PNGase F-digestion (Figure 11).

In summary, the analysis of Nid1, coexpressed with dGlcAT-S or dGlcAT-P in CHO-Lec2-cells, confirms the results from overexpression of the glucuronyltransferases in S2-cells.

## 3. Experimental Section

### 3.1. Materials

All chemicals were purchased from Sigma-Aldrich and were of the highest grade available. Exceptions are indicated in the text.

### 3.2. Cell Culture

Serum-free cell culture of Drosophila S2 cells was performed as described previously [2]. Growth media were Drosophila SFM (Invitrogen, Waltham, MA, USA) and Insectomed SF express (Biochrom, Cambourne, UK) supplemented with 5 μg/mL PS. Transfection and expression of the constructs in S2 cells was performed as described previously [2].

CHO-Lec2 cells were grown in DMEM/HAM’s F12 containing 10% FCS and 5 μg/mL PS at 37 °C and 5% CO2. CHO cell transfection was performed with Attractene Transfection Reagent (Qiagen) according to the suppliers’ protocol. Selection of stable transfectants was achieved by the addition of 10 μg/mL puromycin or 500 μg/mL geneticin G418 to the medium.

Cultivation of the M6749 hybridoma clone was performed in serum-free hybridoma medium ISF-1 (Biochrom) following standard protocols.

### 3.3. Expression and Isolation of Recombinant Glycosylation Probes

The expression vectors encoding the fusion proteins nidogen-1 G1-G2, hDG5 and MUC1VH are described elsewhere [2,17,18]. The recombinant fusion proteins were purified by IMAC (Ni-NTA fast flow, Amersham) from the cell-culture supernatant of S2 respectively CHO-Lec2-cells. Cell culture supernatant was dialyzed against binding buffer (50 mM NaH_2_PO_4_, 300 mM NaCl, 10 mM imidazol, pH 8.0) and filtered before applying onto a PD10 column with equilibrated IMAC material. The supernatant was applied 2 times using gravity flow before washing the column with 50 column volumes binding buffer. Bound protein was eluted with a gradient of 20–200 mM imidazol in PBS. The eluate was concentrated by ultrafiltration.

### 3.4. Cloning of a New Vector Series for Stable Drosophila Cell Transfection

In order to produce homogenous *Drosophila* cell populations that stably overexpress dGlcAT-P or dGlcAT-S, a novel vector series with integrated blasticidin resistance was generated. To this end, the Blast^R^ expression cassette (copia promoter-blasticidin S desaminase cDNA-SV40 polyadenylation signal) was isolated from pCoBlast (LifeTech, Darmstadt, Germany) and transferred to the backbone of the pAc5.1/V5-His A expression vector (LifeTech) as follows. First, pCoBlast was digested with Acc65I (located 3' of the SV40 poly A) followed by a fill-in reaction of the overhangs with Klenow DNA-Polymerase (NEB) according to the manufacturers’ protocol. Then, the Blast^R^ cassette was released from pCoBlast by BamHI digestion (located 3' of the copia promoter) and inserted into pAc5.1/V5-His A cut with BglII (compatible to BamHI at the 5'-end of Blast^R^) and SmaI (blunt end compatible to the blunt ended 3'-end of the Blast^R^ cassette). The resulting expression vector was named pAc5BlastV5HA. In a similar fashion, insertion of the Blast^R^ cassette into pAc5.1/V5-His B and pAc5.1/V5-His C provided pAc5BlastV5HB and pAc5BlastV5HC that have the multiple cloning sites in the two remaining reading frames relative to the coding sequence of the *C*-terminal tags.

In order to eliminate the 6× His-tag while keeping the V5-tag of the novel pAc5BlastV5HA-C expression vector series, each of the vectors was digested with AgeI and PmeI. This was followed by removal of the 5'-overhangs with Mung Bean nuclease (NEB) and relegation according to the manufacturers’ protocols. The procedure generated new expression vectors carrying a multiple cloning site in the three reading frames relative to the C-terminal V5-tag. The novel 6× His-tag deleted vectors were named pAc5BlastV5A, pAc5BlastV5B and pAc5BlastV5C, respectively.

### 3.5. Recombinant Expression of the Glucuronyltransferases

#### 3.5.1. Drosophila GlcAT-P

The cDNA of dGlcAT-P was obtained by PCR using the EST clone LD23788 (Drosophila Genomics Resource Center). For expression of a secreted form of the enzyme, a cDNA-Fragment was amplified that encodes dGlcAT-P sol from Ser31 to Pro443. The flanking restriction sites EcoRI (5') und NotI (3') were added by PCR using the primer pair GTP2 (5'-CGAGAATTCCCGTGAACCCTTCGCCGC-3') and GTP7 (5'-CGAGCGGCCGCACCAGTAGCCTATC-3'). This shortened cDNA was inserted into the EcoRI and NotI sites of version A of pMT/BiP/V5-His (LifeTech), thereby generating an expression cassette for a fusion protein of the *N*-terminal *D. melanogaster* BiP (Hsc70-3) secretion signal and soluble dGlcAT-P fused at the *C*-terminus to a V5-tag for immunological detection and to the 6 × His-tag for affinity purification.

The dGlcAT-P cDNA comprises a natural EcoRI restriction site located 29 base pairs upstream of the start codon. This restriction site was used for the 5'-end in constructs encoding the full-length glycosyltransferase. The cDNA was amplified by PCR using GTP1 (5'-GCTGCAAGCGCAACAGGACG-3') located upstream of the natural EcoRI site and GTP7 (5'-CGAGCGGCCGCACCAGTAGCCTATC-3') and cloned into the EcoRI and NotI sites of pAc5BlastV5A. The obtained expression vector encodes full-length dGlcAT-P in V5-tagged form.

#### 3.5.2. Drosophila GlcAT-S

The cDNA of GlcAT-S was amplified by PCR from the EST clone GH16433 (Drosophila Genomics Resource Center). For expression of a soluble enzyme form, the cDNA encoding Ser42 to Ser479 of dGlcAT-S was obtained by PCR using GTS2 (5'-CGAGAATTCGGAGGAGGGATCTCAC-3') and GTS8 (5'-CGAGCGGCCGCTAAGAATTTTGGAGTGTG-3'). The cDNA was cloned into version B of pMT/BiP/V5-His via the EcoRI and NotI restriction sites to generate a soluble fusion protein with a C-terminal V5- and a 6 × His-tag as described above for dGlcAT-P.

For vectors encoding the full-length glycosyltransferase, the cDNA was amplified using GTS1 (5'-CGAGAATTCACGCAGCTGCAGCAAC-3') and GTS8 and cloned into the EcoRI and NotI sites of version B of pAc5BlastV5. The obtained expression vector encodes full-length dGlcAT-S with a *C*-terminal V5-tag (Figure 1).

#### 3.5.3. CHO-Lec2

For recombinant expression in CHO-Lec2 cells the DNA of the V5-tagged or untagged full length transferases were subcloned into the mammalian expression vector pCDNA3.1 with the restriction endonucleases EcoRI-HF and BamHII-HF (NEB). Correct insertion of the DNA was verified by DNA-sequencing (Seqlab).

### 3.6. Immunofluorescence

Immunostaining of CHO-Lec2 cells was done according to [19], using anti-Giantin (Abcam) and anti-V5 (Invitrogen) according to the manufacturer’s instructions.

### 3.7. Purification of the Recombinant Glucuronyltransferases from Cell Culture Supernatant

The transferases were isolated from the supernatant as described for the recombinant fusion proteins with minor changes. Elution was done with 500 mM imidazole in 300 mM NaCl, 50 mM MES pH 6.5.

### 3.8. Enzymatic Digestions

Endonuclease digestions were done according to the manufacturers’ protocol. Trypsin (Promega, Fitchburg, WI, USA) digestion was done in 50 mM NH4HCO3 at 37 °C overnight. PNGase F (NEB) digestion for subsequent western-blot analysis was performed according to the manufacturers’ protocol.

β-Glucuronidase (Roche, Basel, Switzerland) digestion was achieved during 2 h at 37 °C in 100 mM BisTris pH 6.5.

### 3.9. In-Vitro Glucuronyltransferase Assay

The assay was performed according to [7] at 37 °C using asialo-fetuin as acceptor substrate. Briefly, 50 mM MES pH 6.5, 10 mM MnCl2, 10 mM CaCl2, 1 nM protein and 15 µM UDP-GlcA are added to 10 µL of the enzyme preparation. The sample was incubated for 3 h at 37 °C.

For enzyme purification testing, the standard reaction mixture for dGlcAT-P und -S contained 100 mM MES-NaOH pH 6,5, 10 mM MnCl2, 5 mM Benzyl 2-acetamido-2-deoxy-3-*O*-β-d-galactopyranosyl-α-d-galactopyranoside (Sigma, St. Louis, MO, USA), 0.2 mM UDP-GlcUA (4000 cpm/nmol) and 1–5 μL of the enzyme preparation in a final volume of 50 μL. The reaction was incubated for 20 min at 37 °C in a shaker and was stopped by addition of 450 μL ice-cold ddH2O. The reaction products were quantified by scintillation counting after chromatography on Dowex 1 × 8.

### 3.10. Purification of Monoclonal Antibodies

The hybridoma supernatant containing the antibody M6749 was dialyzed against binding buffer and bound to a Protein l-agarose column. Elution was achieved with glycerol pH 2.7. The eluate was immediately neutralized with Tris-HCl pH 9.0. 114-2G11-A was used directly as hybridoma-supernatant diluted in 2.5% BSA/TBST.

### 3.11. SDS-Polyacrylamide Gel Electrophoresis (SDS-PAGE) and Western Blot

Proteins were separated by 10% or 4%–12% SDS-PAGE in a Mini cell and stained with Coomassie or silver or transferred to a nitrocellulose membrane (Schleicher and Schuell) using wet blot procedure (Mini Protean Cell, BioRad). The protein transfer was performed by 120 mA overnight at 4 °C.

Membranes were blocked in 3% BSA (mAbs anti-tetra-His (Qiagen), M6749 (H. Tanaka, Kumamoto University, Kumamoto, Japan), 114-2G11-A (R. Hokke, LUMC Leiden, The Netherlands)) or 3% skim milk powder (mAb anti-V5 (Invitrogen)) in TBST (TBS containing 0.1% Tween-20) for 1 h and subsequently incubated in the same buffer containing the mAbs (M6749 and 114-2G11-A in 1:50 dilution, 1:5000 dilution of anti-V5 and anti-tetra-His) overnight at 4 °C. After three washes in TBST, HRP-conjugated rabbit anti-mouse Ig (Dako, Glostrup, Denmark) was applied.

1:2000 in 0.5% BSA or 1% skim milk powder in TBST for 1 h. After four washes in TBST and TBS, antibody complexes were detected using the Lumi-Light ECL peroxidase substrate (Roche Diagnostics).

### 3.12. Immunoprecipitation Experiments

Proteins from 200 µL S2-cell lysates in PBS containing 1% Chaps and 2 mM EDTA were immunoprecipitated with mAb 114-2G11-A or HH8 and Protein L-Agarose (Santa Cruz) according to the manufacturers’ instructions. The lysate was precleared with 20 µL Protein l-Agarose under agitation at 4 °C overnight and 2.5 mL of mAb 114-2G11-A or HH8-containing hybridoma supernatant or non-relevant murine IgM (BC3, anti-MUC1, provided by the ISOBM TD-4 Internation Workshop on Monoclonal Antibodies against MUC1, 1996) was coupled to 20 µL Protein l-agarose likewise. Immunoprecipitation was done under the same conditions with the precleared lysates and the antibody-conjugated beads. After 5 washing-steps with PBS, proteins were eluted in 40 µL 2-× non-reducing SDS-sample buffer.

Enrichment of glycopeptides prior to MALDI-MS/MS analysis was achieved by microscale cotton HILIC SPE microtips according to [20].

### 3.13. Protein Identification by Mass Spectrometric Proteomics

Protein identification was done out of SDS-gel bands. Proteins were reduced and alkylated followed by tryptic digestion with 0.1 U trypsin or GluC (Promega, Mannheim, Germany) per 10 µg protein in 0.1 M NH_4_HCO_3_ + 1 mM CaCl_2_ (GluC) for 16 h at 37 °C and LC-MS/MS analysis of protein fragments. ESI-MS/MS analysis of the tryptic peptides was run on an ESI iontrap, the HCT ultra ETDII PTMDiscovery-System (Bruker) coupled with an online easy-nano-LC system (Proxeon). The sample was separated on an analytical C18 column (75 µm × 10 cm) using gradient runs from 0%–35% acetonitrile in 0.1% TFA during 30 min. Ions were scanned with 8100 amu/sec in a range from *m*/*z* 300 to 2500 in MS mode and *m*/*z* 200 to 3000 in MS/MS mode. MS/MS spectra were generated by CID fragmentation. Spectra were acquired with the Compass 1.3 for esquire/HCT software. Proteins were identified by using a local installation of MASCOT 2.4.1 (Matrix Science Ltd, London, UK). The NCBI database was used. Searches were submitted via Proteinscape 3.0 (Bruker Daltoniks, Bremen, Germany) with the following parameter settings: enzyme “trypsin”, species “Drosophila melanogaster”, fixed modifications “carbamidomethyl”, optional modifications “Methionine oxidation” and missed cleavages “1”. The mass tolerance was set to 0.4 Da for peptide and fragment spectra. Proteins with a score above 90 were taken into consideration to exclude isobaric species. Keratins and trypsin are typical contaminants and are not assigned.

### 3.14. Glycan Analysis of Cell Lysates

The *O*-glycome of cell lysates was analyzed by in-gel-*O*-glycan analysis as described [21].

### 3.15. Glycan Derivatisation for MS

*O*-Glycan chains were released from the protein by reductive β-elimination as described [2]. Briefly, 50 mg of the glycoprotein sample was precipitated with chloroform/methanol and dried by vacuum evaporation. To release oligosaccharides by reductive b-elimination, dry samples were treated with 40 mL of 1 M NaBH_4_ in 50 mM NaOH for 18 h at 50 °C. The reaction was stopped by neutralization with acetic acid on ice and deionized with Dowex 50WX8 (H1-form). The supernatant was saved and the beads washed with 200 µL of water. Combined supernatants were dried by vacuum evaporation and depleted of borate by repeated addition of 50 µL of 1% acetic acid in methanol followed by nitrogen evaporation. *N*-glycans were released by PNGaseF-digestion of the tryptic peptides [22]. Permethylation of glycan chains was performed by sequential incubations of the dry samples with finely powdered NaOH in DMSO and methyliodide as described [23].

### 3.16. Glycan and Glycopeptide Analysis by MALDI-TOF-MS/MS

Matrix-assisted laser desorption ionization (MALDI) mass spectrometry was performed on an UltrafleXtreme instrument (Bruker Daltonics). Tryptic peptides or permethylated glycans (approx. 500 ng) in water or methanol were applied to the stainless steel target by mixing a 0.5 µL aliquot of sample with 1.0 µL of matrix (saturated solution of 2,5-dihydroxy benzoic acid in ACN/0.1% TFA, 1:2, or 4-hydroxy-α-cyanocinnamic acid in ACN/0.1% TFA, 1:1). Analyses were performed by positive ion detection in the reflectron mode. Ionization of co-crystallised analytes was induced with a pulsed Smart-beam laser (accumulation of about 5.000 shots) and the ions were accelerated in a field of 20 kV and reflected at 23 kV. MS/MS of precursor ions was performed in the Post-Source-Decay (LIFT) mode.

## 4. Conclusions

Taken together, the results of this study reveal that *Drosophila* GlcAT-P has high intracellular activity towards *O*- and *N*-glycan chains and is responsible for the formation of the glucuronyl-T antigen and the non-sulfated HNK1-epitope. In contrast, dGlcAT-S shows enzymatic activity *in vivo* only towards *N*-glycans and may act on targets different from those analysed in this study.

## Supplementary Files

Supplementary File 1
