# Supplementary material for: Functional Analysis of the Glucuronyltransferases GlcAT-P and GlcAT-S of Drosophila melanogaster: Distinct Activities towards the O-linked T-antigen
Source: Biomolecules. 2016 Jan 6;6(1):8. doi: 10.3390/biom6010008 (PMC4808802; doi:10.3390/biom6010008)
Supplement: Supplementary File 1 [file biomolecules-06-00008-s001.pdf]

Supplementary Materials: Functional Analysis of the Glucuronyltransferases GlcAT-P and GlcAT-S of *Drosophila melanogaster*: Distinct Activities towards the O-linked T-antigen

Isabelle Breloy [1,†,*], Tilo Schwientek [2,†], Deborah Althoff [1], Marvin Holz [1], Tim Koppen [1], Angelika Krupa [1] and Franz-Georg Hanisch [1]

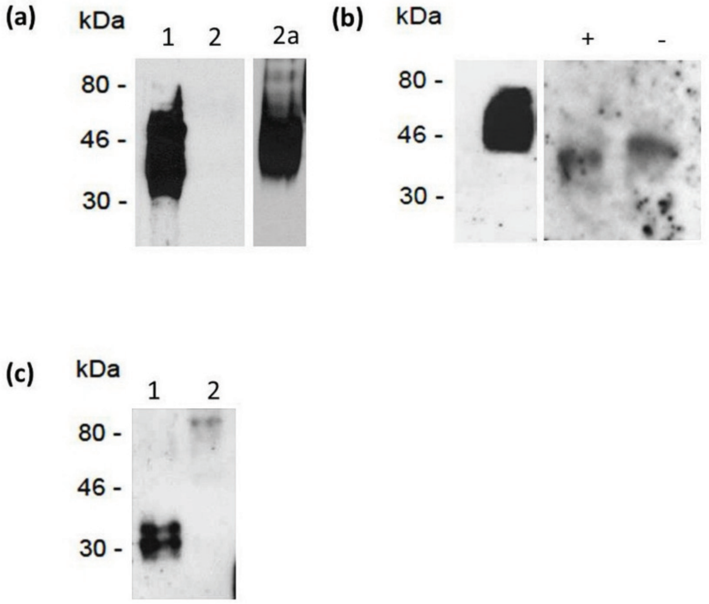

Figure S1. The specificities of the antibodies M6749 and 114-2G11-A were tested using a set of glycoproteins (5 µg/lane) glucuronylated *in vitro* by dGlcAT-Psol. (**a**) Western Blot showing that mAb M6749 is specific for N-linked glucuronic acid. Lane 1, glucuronylated asialofetuin, an *N*-and *O*-glycosylated protein. Lane 2, glucuronylated MUC1VH, an *O*-glycoprotein which is not detected by this antibody. Lane 3, MUC1VH detected by mAb anti-V5; (**b**) Immunoblots demonstrating mAb 114-2G11-A specificity for terminal beta-3-linked GlcA independently of the glycan type. *O*-Glycoprotein MUC1VH can be detected with this antibody (lane 1) as well as *N*- and *O*-glycosylated fetuin before (lane 3) and after (lane 2) PNGaseF digestion. The mass shift of the protein results from successful cleavage of the N-glycans chains; (**c**) MAb 114-2G11-A detects also *N*-glycosylated alpha1-acid glycoprotein after glucuronylation (lane 1). The signal disappears after digestion with beta-glucuronidase (lane2).

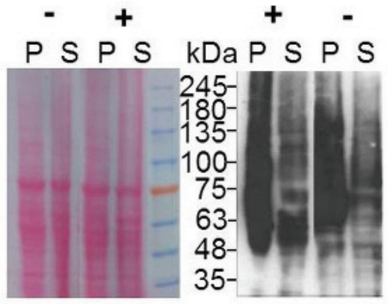

Figure S2. Western Blot with mAb 114-2G11-A (right panel) and loading/transfer control with Ponceau Red (left panel) of cell lysates from dGlcAT-P (P) or dGlcAT-S (S) overexpressing cells before (−) and after (+) PNGase F digestion. Immunostaining reveals a strong increase of GlcA-epitopes in cells overexpressing GlcAT-P in the mass range around 70 kDa. A mass shift due to the cleavage of N-glycans can be observed, but no differences i the anti-GlcA staining patterns or signal intensities are obvious.

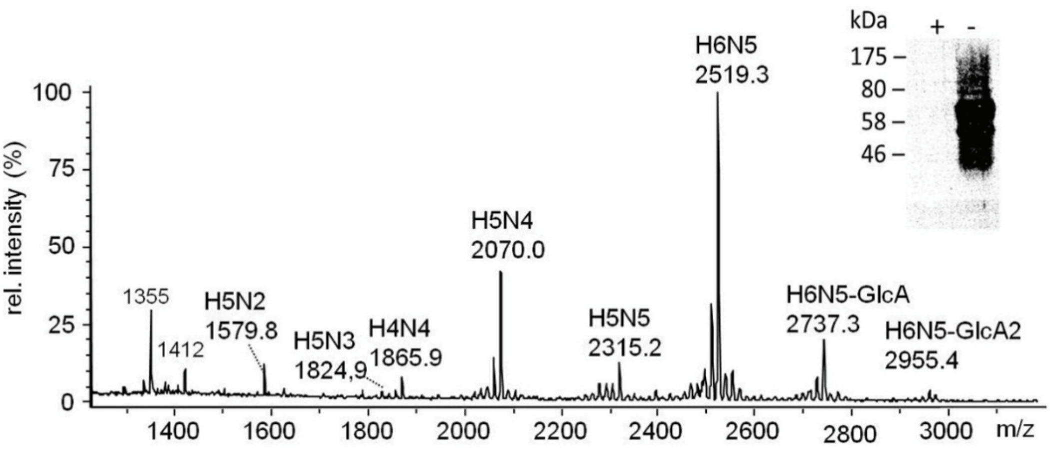

Figure S3. MALDI mass spectrometry of permethylated *N*-glycans after *in vitro* glucuronylation of asialofetuin by dGlcAT-S sol. The positive ion spectrum shows glucuronylated glycan chains at *m*/*z* 2737 and *m*/*z* 2955. Their presence was verified by western blot using anti-HNK1 antibody M6749 (insert) before (−) and after (+) PNGaseF digestion.

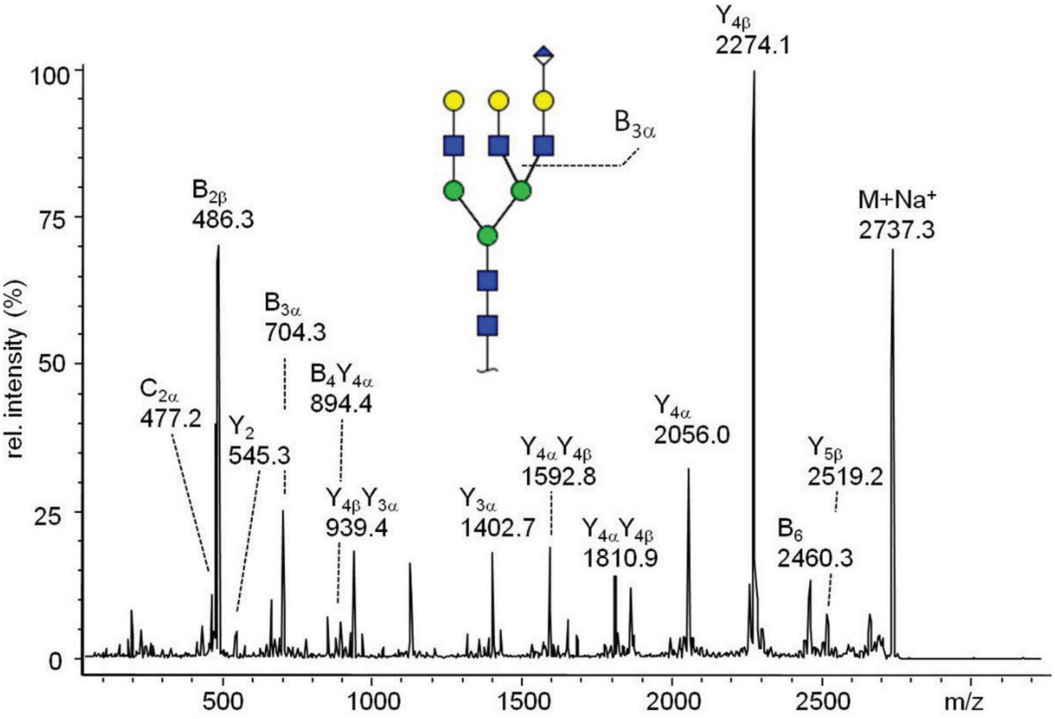

Figure S4. MALDI-MS/MS fragmentation analysis in the Post-Source-Decay mode of the permethylated non-sulfated HNK1-glycan at *m*/*z* 2737 (M + Na^+), which was generated by *in vitro* glucuronylation of asialofetuin with dGlcAT-P sol.

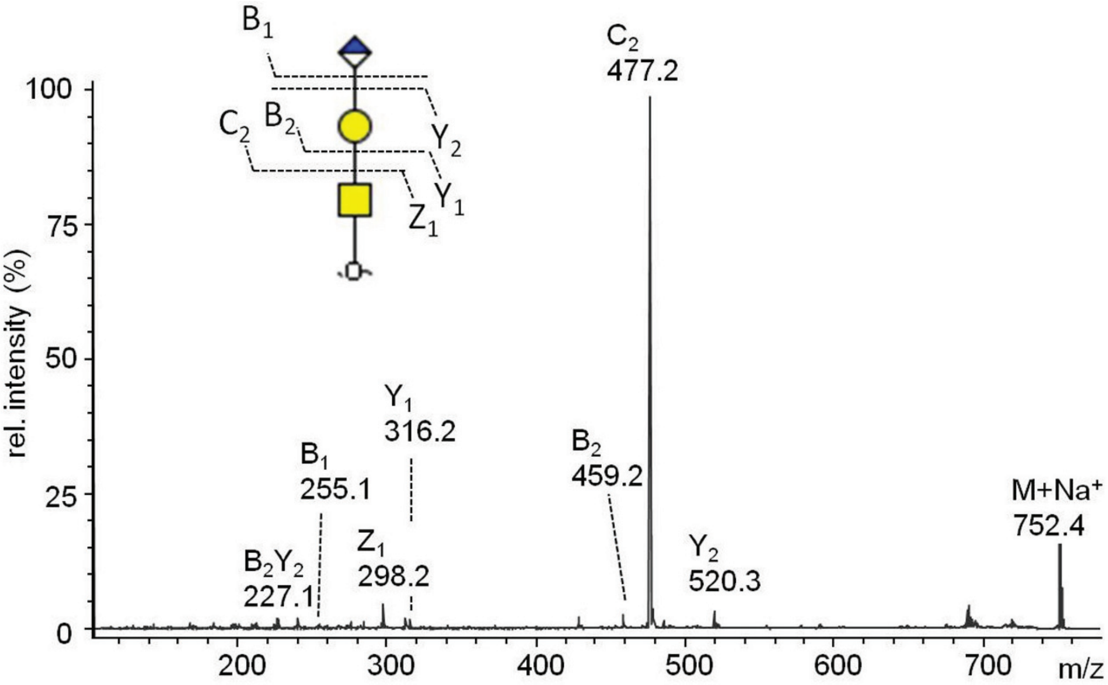

Figure S5. MALDI-MS/MS fragmentation analysis in the Post-Source-Decay mode of the permethylated glucuronyl T-glycan at *m*/*z* 752 (M + Na+) derived from *in vitro* glucuronylated asialofetuin (dGlcAT-P sol). Fragmentation of the glycan is annotated according to the nomenclature of Domon and Costello.

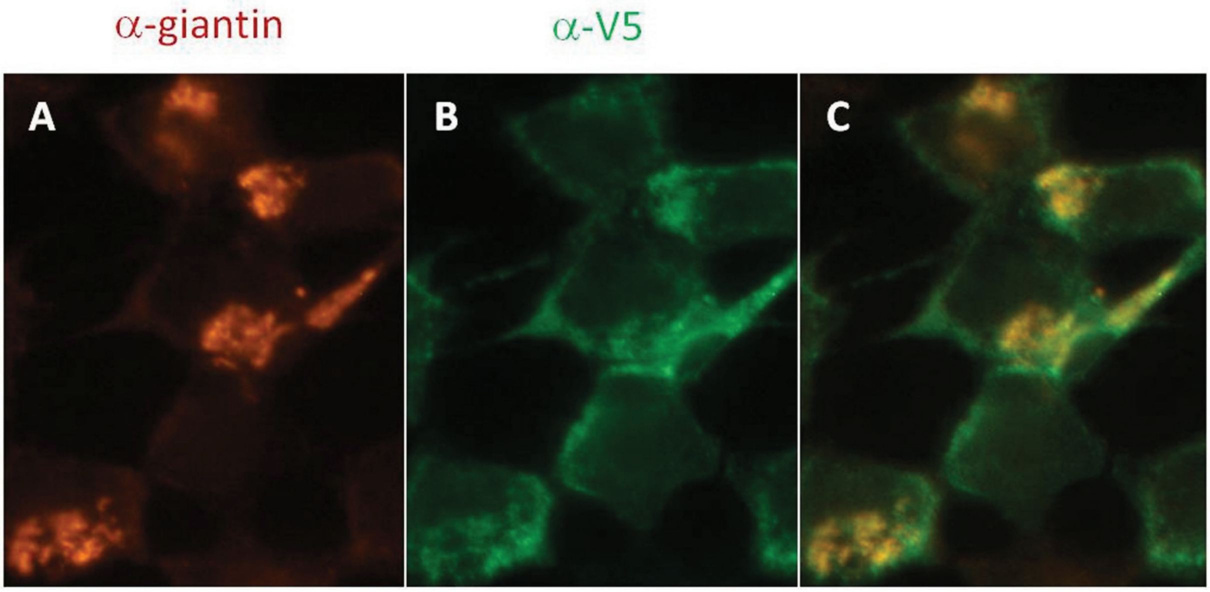

Figure S6. Co-localization of the Drosophila glucuronyltransferases in the Golgi of CHO-Lec2 cells. (**A**) stain of the Golgi membrane with giantin; (**B**) staining of the V5-tagged transferase dGlacAT-S with mAb anti-V5; (**C**) overlay of (**A**,**B**).

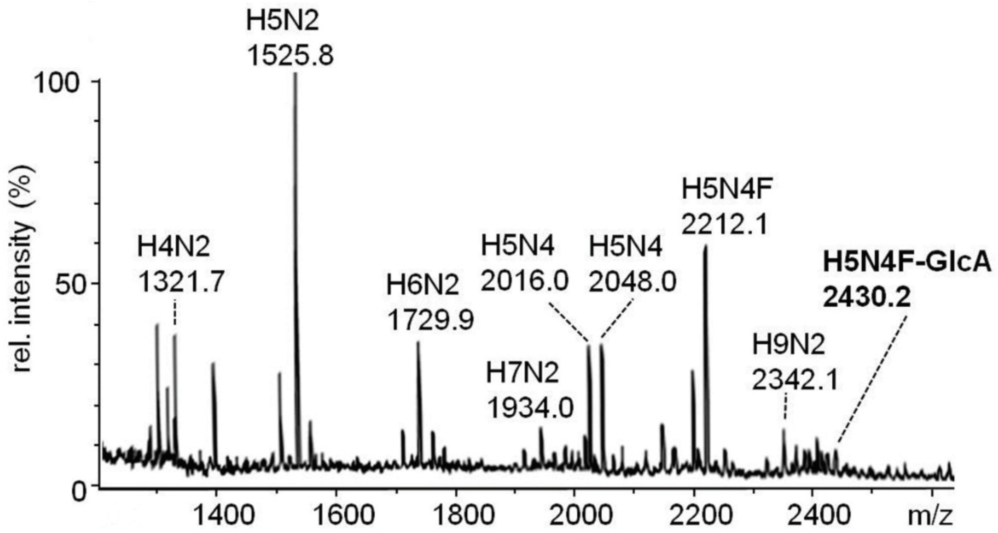

Figure S7. MALDI-MS spectrum of the permethylated N-glycan chains derived by PNGase F digestion from nidogen-1 G1–G2 coexpressed with dGlcAT-P in CHO-Lec2 cells. The monoisotopic masses were detected as M + Na^+-54 or -32 species, corresponding to a loss of sodium-methylate or methanol. A non-sulfated HNK1-epitope was detected at *m/z* 2430 (M + Na^+-32 Da).

Table S1. List of potentially glucuronylated proteins which were identified by mass spectrometry based proteomics of proteins from an S2 cell-lysate, coexpressed with dGlcAT-P and immunoprecipitated with mAb 114-2G11-A. The proteins were separated by SDS-PAGE and extracted from gel slices of the indicated mass range by in-gel-tryptic digestion. (ER: endoplasmatic reticulum, EC: extracellular, N: nucleus, CP: cytoplasm, MI: mitochondrium, MT: microtubule, M: membrane).

Protein	Mascot Score	Identified Peptides	Accession No. (NCBI)	Mass Range
ERp60	1077.09	24	gi|45551086	IP 50–60 kDa
protein disulfide isomerase	940.49	23	gi|17647799	IP 50–60 kDa
Aldehyde dehydrogenase	559.41	12	gi|20129399	IP 50–60 kDa
Chain C, Crystal Structure Of A Filament-like Actin Trimer Bound To The Bacterial Effector Vopl	410.67	9	gi|551702010	IP 50–60 kDa
Ugt86Da glycosyltransferase	358.95	10	gi|21357701	IP 50–60 kDa
heat shock protein 83	344.12	9	gi|17647529	IP 50–60 kDa
peptidase S28	263.37	6	gi|20129649	IP 50–60 kDa
Ugt58Fa	215.98	4	gi|22024248	IP 50–60 kDa
glycoprotein 93	188.39	6	gi|21357739	IP50–60 kDa
vacuolar H[+]-ATPase	128.11	2	gi|17136796	IP 50–60 kDa
oligosaccharide transferase delta subunit	116.13	2	gi|19922486	IP 50–60 kDa
Fimbrin	113.63	2	gi|17647429	IP 50–60 kDa
RE72002p	104.72	3	gi|17944396	IP 50–60 kDa
CG7920	92.83	2	gi|21358615	IP 50–60 kDa
Pyruvate kinase	90.21	2	gi|3108349	IP 50–60 kDa
heat shock protein cognate 72	728.97	17	gi|157658	IP 60–70 kDa
oligosaccharide transferase delta subunit	721.34	14	gi|19922486	IP 60–70 kDa
heat shock protein 83	578.41	11	gi|17647529	IP 60–70 kDa
heat shock protein cognate 4	578.07	4	gi|17737967	IP 60–70 kDa
Chain C, Crystal Structure Of A Filament-like Actin Trimer Bound To The Bacterial Effector Vopl	454.09	11	gi|551702010	IP 60–70 kDa
heat shock protein cognate 71	193.86	4	gi|157667	IP 60–70 kDa
heat shock protein 60	181.86	4	gi|3757828	IP 60–70 kDa
CG2918	164.50	3	gi|20128923	IP 60–70 kDa
hexosaminidase 2	138.87	2	gi|17933586	IP 60–70 kDa
CD98 heavy chain	126.66	3	gi|17945866	IP 60–70 kDa
vacuolar ATPase	116.35	2	gi|17136986	IP 60–70 kDa
Twinstar	115.28	2	gi|17136986	IP 60–70 kDa
glycoprotein 93	107.26	2	gi|21357739	IP 60–70 kDa

Moesin	107.24	2	gi|24640670	IP 60–70 kDa
Inos	96.05	2	gi|17137626	IP 60–70 kDa
heat shock protein cognate 72	1828.28	39	gi|157658	IP 70–80 kDa
heat shock protein cognate 4	858.27	14	gi|17737967	IP 70–80 kDa
heat shock protein 83	328.07	7	gi|17647529	IP 70–80 kDa
heat shock protein cognate 71	271.38	6	gi|157667	IP 70–80 kDa
glycoprotein 93	154.93	3	gi|21357739	IP 70–80 kDa
heat shock protein 83	678.00	15	gi|17647529	IP 80–100 kDa
Actin	588.85	12	gi|156750	IP 80–100 kDa
heat shock protein cognate 72	333.63	8	gi|157658	IP 80–100 kDa
heat shock protein cognate 4	145.05	1	gi|17737967	IP 80–100 kDa
glycoprotein 93	126.20	4	gi|21357739	IP 80–100 kDa
BerH2-scFv-hpRNase	90.90	4	gi|164508020	IP 80–100 kDa
Chain C, Crystal Structure Of A Filament-like Actin Trimer Bound To The Bacterial Effector Vopl	625.09	14	gi|551702010	IP 100–150 kDa
Actin 87E	559.63	1	gi|17137090	IP 100–150 kDa
heat shock protein 83	275.46	4	gi|17647529	IP 100–150 kDa
Na^+, K^+-ATPase	222.01	3	gi|17861704	IP 100–150 kDa
Scavenger receptor class C	112.17	2	gi|984515	IP 100–150 kDa
BerH2-scFv-hpRNase	96.81	4	gi|164508020	IP 100–150 kDa
heat shock protein cognate 72	1408.20	28	gi|157658	60–70 kDa
heat shock protein cognate 4	1050.97	15	gi|17737967	60–70 kDa
protein disulfide isomerase	922.30	22	gi|17647799	60–70 kDa
heat shock protein 83	904.56	20	gi|17647529	60–70 kDa
ERp60	542.46	12	gi|45551086	60–70 kDa
Chain C, Crystal Structure Of A Filament-like Actin Trimer Bound To The Bacterial Effector Vopl	394.68	11	gi|551702010	60–70 kDa
heat shock protein 60	357.71	8	gi|33636453	60–70 kDa
glycoprotein 93	324.11	4	gi|21357739	60–70 kDa
heat shock protein cognate 1	305.17	1	gi|17647515	60–70 kDa
Inos	264.43	6	gi|17137626	60–70 kDa
heat shock protein cognate 71	240.47	4	gi|157667	60–70 kDa
CG2918	199.21	4	gi|20128923	60–70 kDa
vacuolar ATPase	195.54	4	gi|17136986	60–70 kDa
thioredoxin peroxidase 1	170.66	3	gi|17157991	60–70 kDa
elongation factor 1alpha48D	153.74	4	gi|17137572	60–70 kDa
calcium-binding protein 1	131.37	2	gi|19921434	60–70 kDa
eukaryotic initiation factor 4a	123.69	3	gi|17136248	60–70 kDa
aldehyde dehydrogenase	112.62	3	gi|20129399	60–70 kDa
calnexin	110.33	2	gi|2213427	60–70 kDa
beta-1 tubulin	99.21	2	gi|158739	60–70 kDa

© 2016 by the authors; licensee MDPI, Basel, Switzerland. This article is an open access article distributed under the terms and conditions of the Creative Commons by Attribution (CC-BY) license (http://creativecommons.org/licenses/by/4.0/).
